# Do Daytime Activity, Mood at Bedtime and Unit Tumult Predict Nighttime Sleep Quality of Long-Term Care Residents?

**DOI:** 10.1093/geroni/igab046.3191

**Published:** 2021-12-17

**Authors:** Murad Taani, Christine Kovach

**Affiliations:** 1 University of Wisconsin-Milwaukee, Milwaukee, Wisconsin, United States; 2 University of Wisconsin Milwaukee, Milwaukee, Wisconsin, United States

## Abstract

Sleep quality declines in old age and is particularly poor for long-term care (LTC) residents with dementia. Compromised sleep quality is associated with severe cognitive and neuropsychiatric symptoms, agitation, aggressiveness, and poor quality of life. Based on the premise that stressors can have a cumulative effect on people with dementia throughout the day that contributes to negative consequences later in the day, we examined if daytime activity, mood, and unit tumult were associated with sleep quality. A convenience sample of 53 LTC residents with dementia participated in this correlational study. Objective sleep quality and activity variables were measured using Actigraphy, and mood was measured by the Observed Emotion Rating Scale. Unit tumult was defined as events in the residents living area that are deviations from the typical day (i.e., census changes, being cared for by a certified nursing assistant from a temporary staffing agency, and lower than usual staffing level). Comorbid illness and level of dementia were control variables. Half of the sample had a sleep efficiency that was less than .85 and were awake for more than 90 minutes at night. Comorbid illness, negative mood at bedtime, and daytime activity level accounted for 26.1% of the variance in total sleep minutes. Census changes and the use of temporary agency staff were associated with poor total sleep time and sleep efficiency. Findings suggest that daytime activity, mood at bedtime, and unit tumult should be considered when designing and testing interventions to improve sleep quality among LTC residents with dementia.

